# Characterization of a novel inhibitory human monoclonal antibody directed against *Plasmodium falciparum* Apical Membrane Antigen 1

**DOI:** 10.1038/srep39462

**Published:** 2016-12-21

**Authors:** Dominika J. Maskus, Michał Królik, Susanne Bethke, Holger Spiegel, Stephanie Kapelski, Melanie Seidel, Otchere Addai-Mensah, Andreas Reimann, Torsten Klockenbring, Stefan Barth, Rainer Fischer, Rolf Fendel

**Affiliations:** 1 Fraunhofer Institute for Molecular Biology and Applied Ecology IME, Aachen, Germany; 2 Institute for Molecular Biotechnology, RWTH Aachen University, Aachen, Germany; 3 Institute for Applied Medical Engineering at RWTH Aachen University and Hospital, Department of Experimental Medicine and Immunotherapy, Aachen, Germany; 4 Faculty of Allied Health Sciences, Kwame Nkrumah University of Science and Technology, KNUST, Kumasi, Ghana.

## Abstract

Malaria remains a major challenge to global health causing extensive morbidity and mortality. Yet, there is no efficient vaccine and the immune response remains incompletely understood. Apical Membrane Antigen 1 (AMA1), a leading vaccine candidate, plays a key role during merozoite invasion into erythrocytes by interacting with Rhoptry Neck Protein 2 (RON2). We generated a human anti-AMA1-antibody (humAbAMA1) by EBV-transformation of sorted B-lymphocytes from a Ghanaian donor and subsequent rescue of antibody variable regions. The antibody was expressed in *Nicotiana benthamiana* and in HEK239-6E, characterized for binding specificity and epitope, and analyzed for its inhibitory effect on *Plasmodium falciparum*. The generated humAbAMA1 shows an affinity of 106–135 pM. It inhibits the parasite strain 3D7A growth *in vitro* with an expression system-independent IC_50_-value of 35 μg/ml (95% confidence interval: 33 μg/ml–37 μg/ml), which is three to eight times lower than the IC_50_-values of inhibitory antibodies 4G2 and 1F9. The epitope was mapped to the close proximity of the RON2-peptide binding groove. Competition for binding between the RON2-peptide and humAbAMA1 was confirmed by surface plasmon resonance spectroscopy measurements. The particularly advantageous inhibitory activity of this fully human antibody might provide a basis for future therapeutic applications.

Malaria remains a major challenge to global healthcare and is one of the major causes of morbidity and mortality in childhood, especially in Sub-Saharan Africa. Of the six plasmodium species which are pathogenic to humans, *Plasmodium falciparum* is the most frequent. It brings about the severest form of malaria, malaria tropica, affecting mostly children by cerebral malaria and severe malarial anaemia^1^,^2^. Natural occurring premunition to malaria develops slowly and wanes without frequent exposure^2^,^3^,^4^,^5^. Several lines of defense of the human immune system contribute to the successful control of plasmodial infections. Besides cellular mechanisms, *e.g.* by T cell-mediated killing of blood and liver stages^6^,^7^,^8^, the defense mediated by antibodies plays a critical role. The spectrum of anti-plasmodial antibodies usually increases with age, *i.e.* with cumulative exposure^9^,^10^,^11^ and the majority of the antibodies is directed against merozoites^11^, the invasive forms of the erythrocytic replication cycle. Antibodies, representing the adaptive arm of humoral immune defense, are all-round talents which can exert their functions by mere binding (primary function) and by recruiting effector cells and/ or complement factors (secondary functions). In case of plasmodial infections the modes of action during the blood stage thus include (1) the blocking of erythrocyte invasion by merozoites, (2) the neutralization of merozoites by agglutination, (3) the initiation of the complement cascade resulting in further opsonization and lysis, and (4) the recruitment of neutrophilic granulocytes and monocytes/macrophages^12^,^13^,^14^,^15^,^16^,^17^,^18^,^19^,^20^.

The invasion of merozoites into erythrocytes is a complex process, which can be subdivided into a pre-invasion phase, the ‘classical’ invasion phase, and an echinocytosis phase^21^,^22^. One of the key proteins during the ‘classical’ invasion is Apical Membrane Antigen 1 (AMA1). In the human host this protein is mainly expressed in the late plasmodial stages, *i.e.* in late trophozoites and schizonts^23^,^24^,^25^. Initially, the 83-kDa precursor of AMA1 (AMA1_83_) is localized in the micronemes^26^,^27^. By the time of schizont rupture and release of young merozoites AMA1_83_ is processed to give the mature 66-kDa form (AMA1_66_) which remains membrane-bound^24^,^28^,29. AMA1_66_ then translocates to the merozoite’s apical end to fulfill its function in the invasion process by interacting with Rhoptry Neck Protein 2 (RON2)^24^,^27^. RON2 is secreted from the rhoptries just prior to invasion and inserts into the erythrocyte membrane^30^,^31^,^32^. The interaction of AMA1 and RON2 takes place between the hydrophobic trough of AMA1 and a small extracellular hydrophobic domain of RON2^31^,^33^,34. This interaction is critical since the AMA1:RON2 complex is part of the sealing moving junction and constitutes the anchor for the actin-myosin motor which pulls the merozoite into the red blood cell to be invaded^32^,^35^,^36^,^37^. Several studies showed that AMA1-specific antibodies can inhibit invasion^38^,^39^.

Many anti-plasmodial monoclonal antibodies (mAbs) have been generated in mice or other rodents which helped to gain valuable insights into the functions of a plethora of plasmodial proteins^40^,^41^,^42^,^43^,^44^. However, such mAbs do not necessarily reflect the naturally acquired anti-plasmodial immunoglobulin repertoire in humans which takes years – or even decades – to develop. So far, only few human anti-plasmodial monoclonal antibodies (humAbs) have been generated. Among these are humAbs directed at Merozoite Surface Protein 1 (MSP1), MSP2, MSP3, MSP10, NPNA1, Pfs48/45, and VAR2CSA^45^,^46^,^47^,^48^,^49^,^50^,^51^,^52^. However, to the best of our knowledge, no humAb specific to AMA1 has been described yet. Here, we report the isolation, expression and characterization of the first human monoclonal antibody recognizing *P. falciparum* AMA1, called humAbAMA1.

## Results

### Selection of PBMC donor and EBV-transformation and screening

**Figure 1:**
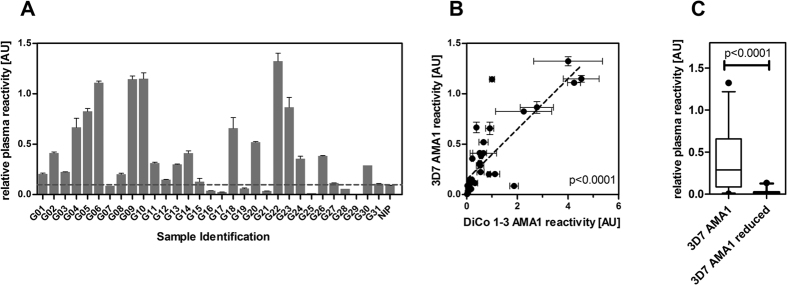
Plasma IgG reactivities against AMA1. Using samples from 31 Ghanaian blood donors (G01–31) as well as a European control pool of two donors which had never been exposed to Malaria (NIP), ELISA was performed to estimate the reactivity against 3D7 AMA1. As positive control, a pool of four highly reactive Ghanaian donors was used. Values reflect relative reactivity in comparison to this positive control’s reactivity, which was set to 1 (**A**). The reactivity of the samples to AMA1 (3D7) and AMA1 (DiCo 1-3) (previously published in Feller *et al*.)^54^ were compared and the degree of correlation was estimated using the Spearman rank sum test (**B**). The reactivity of the plasma samples against both the native antigen and the reduced/alkylated antigen was estimated by ELISA, and shown as a box plot (**C**). The p-values were estimated using the Wilcoxon matched pairs test.

In order to choose a promising candidate for the generation of an AMA1-specific human monoclonal antibody, plasma of 31 adult Ghanaian blood donors were screened by indirect ELISA for IgG reactivity against different recombinant variants of AMA1, including the allelic variant of Plasmodium falciparum strain 3D7, as well as a mixture of three artificial diversity covering variants of AMA1 (described by Remarque *et al*.^53^). Against the single allele recombinant antigen AMA1 (3D7), 23 out of 31 (74%) of the Ghanaian plasma samples showed a positive immune response (Fig. 1A). The reactivity of the plasma against AMA1 (DiCo1-3) has been shown before^54^. Here, 30 out of 31 samples showed a positive reaction (reactivity superior of the negative control plus 2 standard deviations), corresponding to 97%.

The reactivities of the Ghanaian plasma samples against the variants AMA1 (3D7) and AMA1 (DiCo1-3) showed a strong positive correlation (p < 0.0001, Fig. 1B). Next, these plasma IgG were tested for recognition of conformational epitopes of AMA1. Therefore, ELISA using the same plasma samples were carried out similarly, but additionally using reduced/alkylated version of AMA1 (3D7). The results indicate that the majority of the antibodies are directed against conformational epitopes. The median reactivity of the plasma samples to reduced vs. the reactivity to the native antigen decreased by a factor of 17 (p < 0.0001, Wilcoxon matched pairs test, Fig. 1C). Only 2 of the 31 samples showed a reactivity against the reduced antigen AMA1 (3D7) and reactivities of these two were only slightly above the threshold of two standard variations above the negative control reactivity.

The sample of donor G10 had the highest reactivity against AMA1 (DiCo1-3) and is also among the samples with highest reactivity against AMA1 (3D7). This is why PBMCs of donor G10 were selected for the following steps of antibody generation. The PBMCs were thawed, antigen-specific B-cells were stained (IgG^+^/CD22^+^/anti-AMA1 (DiCo1-3)^+^) and sorted by flow cytometry. The supernatants of the cultured lymphoblastoids were tested for specific antibody production after two weeks of culture, the supernatant of well 1E4 showed high reactivity against AMA1 (DiCo1-3). Subsequently, the sequences coding for variable domains of the antibody light and heavy chain were amplified from cDNA from these cells, sequenced and cloned into expression vectors for recombinant production.

### Recombinant expression of humAbAMA1

**Figure 2:**
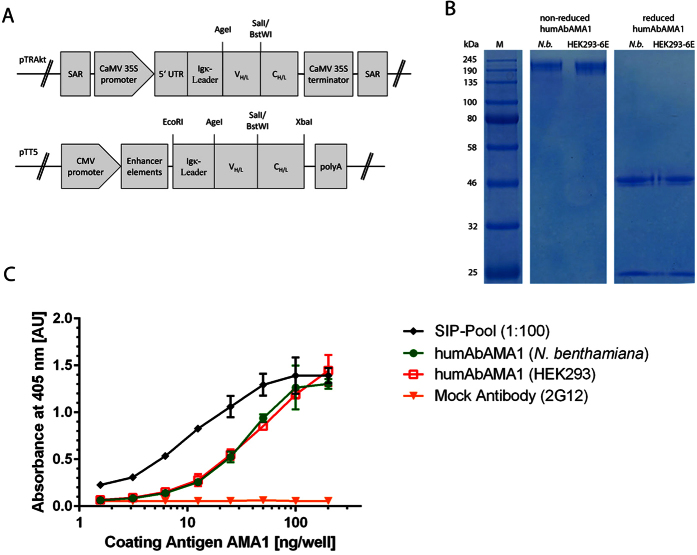
Cloning, expression and binding specificity of humAbAMA1. HumAbAMA1 was cloned as full length expression construct in both vectors pTRAkt and pTT5 for transient expression in plants and in HEK293-6E cells, respectively (**A**). The expression and purity of the full length antibodies expressed both in plants (*N. benthamiana*) and in mammalian cells (HEK293) was controlled by SDS-PAGE. Both non-reduced and reduced samples of the antibodies were loaded on the gel (**B**). The binding of humAbAMA1to AMA1 (3D7) was tested by indirect ELISA. Dilutions of the antigen (AMA1 (3D7)) were coated onto ELISA plates, then human antibodies were applied followed by goat anti-human antibody coupled to alkaline phosphatase (Dianova, Hamburg, Germany). Black diamonds: Semi-immune (premune) plasma pool; green circles: humAbAMA1 (*N. benthamiana*); Red open squares: humAbAMA1 (HEK293); orange inverted triangles: mock control antibody (2G12).

The expression cassettes for the antibody production in plants and mammalian cell culture are shown in Fig. 2A. HumAbAMA1, the recombinant antibody of EBV-clone 1E4, was produced as a full-size human IgG1:κ by *Agrobacterium*-mediated transient protein expression in *N. benthamiana* plants. After harvest and homogenization of the plant material, the antibodies were purified by Protein A chromatography. The final yield of the pure and intact plant produced antibody was 70 mg/kg fresh leaf material. Similarly, secretory production of the antibody was performed by transient transfection of HEK293-6E cells. The cell culture supernatants were purified by Protein A chromatography. Here, the productivity of the antibody after 6 days of antibody production was approx. 10 mg/l cell culture supernatant. Integrity and purity of both antibodies was assessed by SDS-PAGE (Fig. 2B).

### HumAbAMA1 recognizes a conformational epitope and binds to it with high affinity

**Figure 3:**
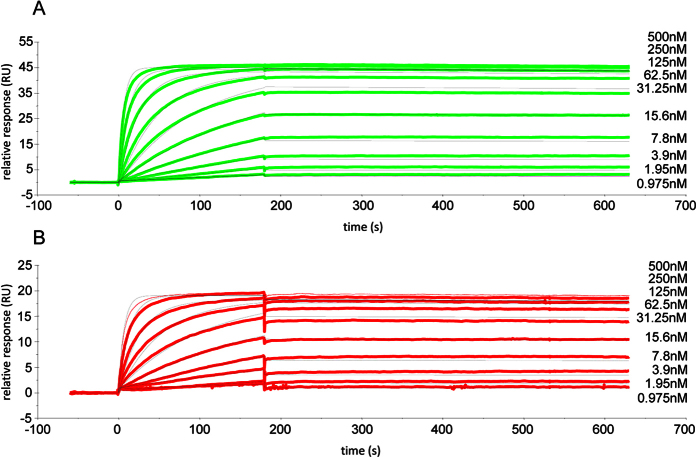
Sensorgrams of surface plasmon resonance (SPR) measurement for the determination of humAbAMA1 affinity to AMA1 (3D7). The affinity of the antibody humAbAMA1 was estimated by SPR spectroscopy. For each cycle recombinant and purified humAbAMA1 either produced in *N. benthamiana* (**A**, green sensogramms) or produced in HEK293-6E cells (**B**, red sensograms) was captured on a Protein A surface (65 RU for *N. benthamiana* produced or 32 RU for HEK produced humAbAMA1). Subsequently, the antigen AMA1 (3D7) was injected at concentrations of 500 nM, 250 nM, 125 nM, 62, 5 nM, 31.25 nM, 15.6 nM, 7.6 nM, 3.9 nM, 1.95 nM and 0.975 nM for 180 s, followed by a buffer injection step for 420 sec. To determine kinetic constants, the data was fitted using a 1:1 Langmuir interaction model using the software BIAEval 4.0. Fit curves are shown in light grey.

The binding specificity of the antibody humAbAMA1 was first tested in indirect ELISA. The reactivity of the antibody was concentration dependent, with a detection limit of approx. 6 ng/well (Fig. 2C). AMA1 possesses eight disulfide bridges, which strongly stabilize its tertiary structure^55^. Rodent-derived anti-AMA1 mAbs, such as the murine mAb 1F9 and the rat mAb 4G2 recognize conformational, reduction-sensitive epitopes^43^,^56^. This is also the case for humAbAMA1, which does not react with reduced/alkylated AMA1 in indirect ELISA (data not shown). Surface plasmon resonance (SPR) spectroscopy measurements were used to compare the affinity of humAbAMA1 produced in plants and in HEK293-6E cells towards the recombinant antigen AMA1 (3D7), revealing very high affinities (K_D_) of 135 ± 8 pM and 106 ± 5 pM for the plant and HEK-derived antibodies, respectively (Fig. 3). Additionally, the affinity of the antibody humAbAMA1 produced in *N. benthamiana* against two further AMA1 antigen variants representing the strains HB3 and FCR3 have been measured. The binding towards AMA1 (HB3) is in the similar range (667 ± 7 pM), whereas the binding to AMA1 (FCR3) is two orders of magnitude lower (73 ± 0.2 μM). The kinetics of the antibodies are more precisely summarized in Table 1.

### HumAbAMA1 recognizes AMA1 in its native context on the surface of merozoites and schizonts

**Figure 4:**
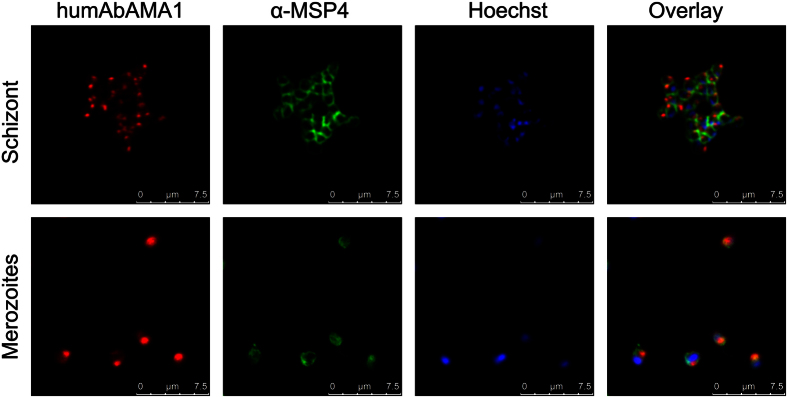
Immunofluorescence imaging of humAbAMA1. The specific binding of the recombinant antibody humAbAMA1 to both schizonts (upper panel) and free merozoites (lower panel) was analyzed by immunofluorescence assays. Parasites were co-stained with humAbAMA1 (red, first column), murine anti-MSP4 antibody (green, second column) and the nuclear stain Hoechst 33342 (blue, third column). The last column shows the overlay image. Human and murine antibodies were detected by fluorescently labelled antibodies goat-anti-human IgG (H + L)-Cy3 (Dianova) and goat-anti-mouse IgG (H + L)-Alexa Fluor^®^ 488 (Life Technologies), respectively. Images were taken at a 630-fold magnification with a Leica TCS SP8 microscope. The right panel shows the overlay of the three fluorescence images. Scale bar 7.5 μm.

Next, humAbAMA1 was tested for binding native AMA1 located on merozoites and schizonts. To this end, immunofluorescence assays (IFAs) were carried out using double staining with the humAbAMA1, as well as the MSP4-specific antibody 2.44 (Fig. 4, upper panel). We observed a characteristic punctiform staining at one end of ripening and free merozoites, which is in line with the apical localization of AMA1. In some cases we also saw a staining of the outer membrane of free merozoites, indicating colocalization with antibody 2.44 (Fig. 4, lower panel). This is consistent with the partial redistribution of AMA1 once the merozoites have been released upon schizont rupture^57^. The data obtained strongly resemble immunofluorescence images using AMA1-specific antibodies published earlier^38^,^57^.

### *Plasmodium falciparum* strains 3D7A, HB3 and FCR3 are strongly inhibited by humAbAMA1

**Figure 5:**
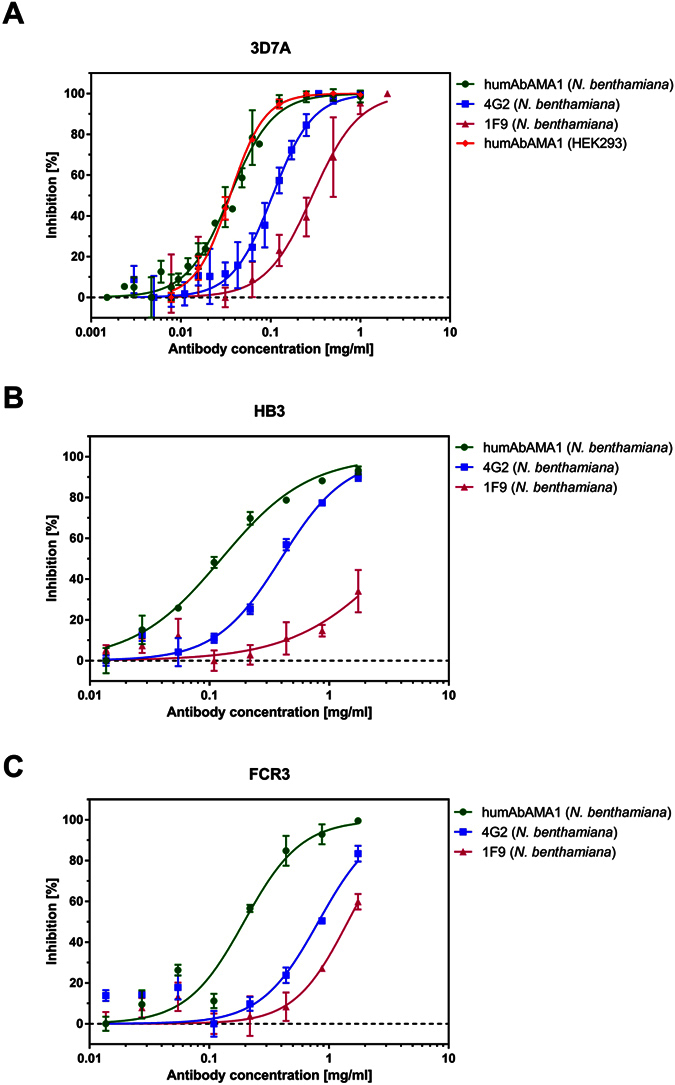
*In vitro* growth inhibition activity of humAbAMA1. The anti-parasitic effect was estimated using a standardized Growth Inhibition Assay (GIA). Purified antibodies were added to the parasite strain *Plasmodium falciparum* 3D7A (**A**), HB3 (**B**) and FCR3 (**C**) at schizont stage and co-incubated for 40–46 h. Growth of the parasites was estimated by a pLDH assay. All tests were carried out in technical triplicates; error bars correspond to the standard error of the mean. Antibodies tested were the humAbAMA1 produced in plants (green circles), humAbAMA1 produced in HEK293 (red open squares, only tested in strains 3D7A), chimeric antibody 4G2 (blue closed squares) and chimeric 1F9 (purple triangles).

The inhibitory potential of humAbAMA1 was tested in *in vitro* growth inhibition assay (GIA) with *P. falciparum* 3D7A, HB3 and FCR3. We found that *P. falciparum* strain 3D7A was reproducibly inhibited by plant-produced humAbAMA1 with an IC_50_ value of 35 μg/ml (95% confidence interval (CI): 33–37 μg/ml). Inhibition assays using the antibodies produced in mammalian cell culture showed the same IC_50_ value of 35 μg/ml (95% CI: 34–37 μg/ml) (Fig. 5A). As controls, we expressed the rodent-derived antibodies described in the literature (4G2 and 1F9) as chimeric antibodies. The IC_50_ values of these antibodies were 105 μg/ml (95% CI: 99–111 μg/ml) and 292 μg/ml (95% CI: 250–342 μg/ml) for the chimeric antibodies 4G2 and 1F9, respectively, thus being three to eight times higher than the IC_50_ value of humAbAMA1. The IC_50_-value of the humAbAMA1 against the parasite strains HB3 and FCR3 was also 3-fold and 4-fold lower than the chimeric antibody 4G2, respectively. The second chimeric control antibody inhibited the parasite strains HB3 and FCR3 at values above 1000 μg/ml, thus the estimated values were 7 to >13-fold higher than the antibody humAbAMA1. The assays are represented in Fig. 5 and the estimated IC_50_-values summarized in Table 2.

**Figure 6:**
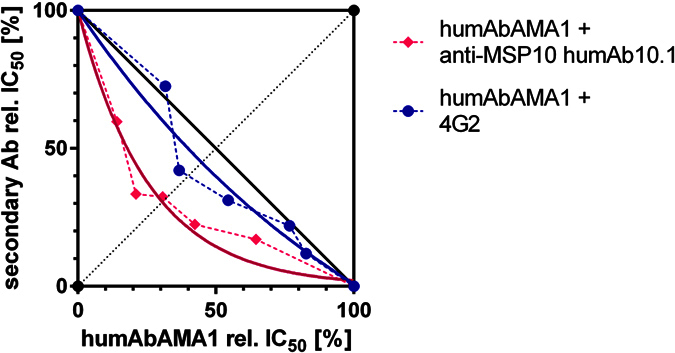
Synergistic effect of monoclonal antibodies on parasitic growth inhibition. The combinations of the humAbAMA1 with either the MSP10-specific humAb10.1 (pink diamonds) or the chimeric antibody 4G2 (blue circles), respectively, were tested for synergistic growth inhibition. Antibodies were applied singly as well as in combinations. Antibodies were mixed in different ratios (1:4, 1:2, 1:1, 2:1, 4:1) proportional to their respective estimated IC_50_ values. Titrations of each of the mixtures were added to the parasites. The IC_50_ value of each of the antibody mixes was estimated and is depicted as relative inhibition. Curves were fitted using the One phase Decay function of GraphPad Prism 5.0.

Next, combinations of the human monoclonal antibodies humAbAMA1 and humAb10.1 (anti-MSP10, previously isolated in our laboratory)^52^ were tested for synergistic effects. The antibodies were mixed in five different ratios, added in serial dilutions to the parasite culture (*P. falciparum* strain 3D7A) and the IC_50_ of each combination was determined. Generally, two calculations are performed to evaluate the degree of synergy, the sum of FIC and HEWLETT’s S. Values corresponding to sum of FIC below 1 and HEWLETT’s S above 1 are regarded as synergistic, FIC below 0.5 and HEWLETT’s S above 2 reflect strongly synergistic effects. As shown in the isobologram, the combination of the AMA1-specific antibody and the MSP10 antibody humAb10.1 shows synergistic inhibition of the parasite strain 3D7A (Fig. 6). The calculated FIC and HEWLETT’s S are 0.68 and 1.64, respectively. In comparison, two AMA1-specific antibodies (humAbAMA1 and 4G2) rather act in an additive way, as shown in Fig. 6 (sum of FIC = 0.92 and HEWLETT’s S = 1.11).

### HumAbAMA1 partially shares an epitope with mAb 1F9

**Figure 7:**
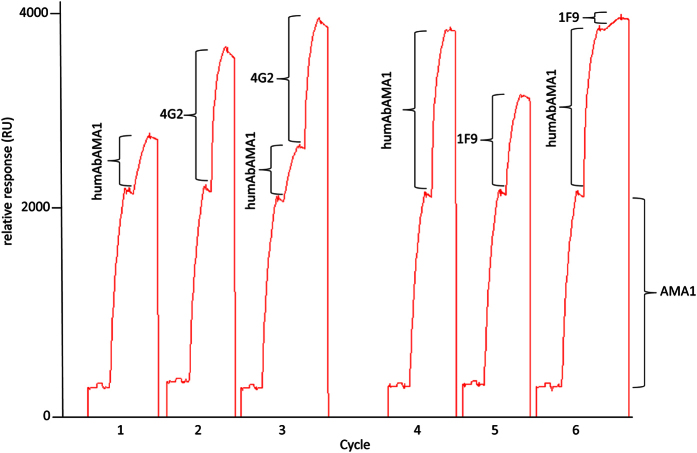
Competition measurements of humAbAMA1 with the 4G2 and 1F9. Using SPR spectroscopy, pairwise epitope mapping was performed. The chip was functionalized with antibody mAb1D7, which binds to domain III of AMA1. This chip was regenerable. Subsequently, in each cycle AMA1 (3D7) (1750 RU) was caught. Then, humAbAMA1, 1F9, or 4G2 were injected singly (cycles 1, 2, 4 and 5) or consecutively (cycles 3 and 6). In cycles 1–3, 1 μg/ml of each antibody was applied, whereas in cycles 4–6, the concentration of each antibody was 10 μg/ml. In case spectral resonance is increasing with the binding of the secondary antibody, no competition is detectable, in case the signal is not increased after binding of the secondary antibody, the epitopes either overlap or binding is compromised by allosteric competition.

**Figure 8:**
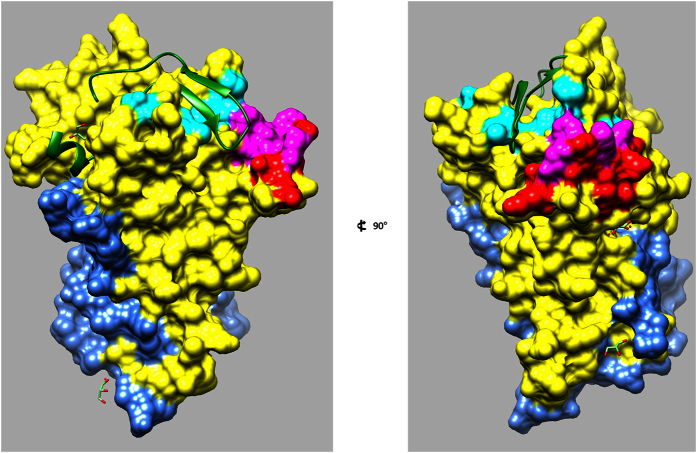
Epitope Mapping. The epitope of humAbAMA1 was mapped using the CLIPS-constrained peptides. Its localization pertains to domain I of AMA1 (yellow) and is highlighted in red. The published epitope of 1F9 within domain I is highlighted in cyan, the region in which the epitopes of humAbAMA1 and 1F9 overlap is highlighted in magenta^56^. The domain II of AMAI is shown in blue. The RON2 peptide (green) is bound to its specific hydrophobic binding groove in domain I. The structural data was obtained from the protein data base PDB, accession number 3ZWZ^102^. The structure in the second image is a 90° rotation of the first image.

After the confirmation of the strong inhibitory response in GIAs, humAbAMA1 was tested for potential epitope overlapping with the well-characterized inhibitory antibodies 1F9 or 4G2. To this end, a “pairwise epitope mapping” was carried out. In a multiple capture setup AMA1 (3D7) was first caught on a mAb1D7 (AMA1 domain III-specific antibody) surface. Then, the antibodies to be tested were injected singly or consecutively. It was found that humAbAMA1 and 1F9 partially share an epitope, *i.e.* 1F9 could not bind efficiently anymore if humAbAMA1 had been injected first. However, their epitopes appeared not to be completely identical since a small proportion of 1F9 could still recognize AMA1 (3D7) (Fig. 7, cycle 6). In cycle 3 (Fig. 7), it became clear that humAbAMA1 and 4G2 could bind simultaneously. Thus, their corresponding epitopes neither overlap nor show signs of allosteric competition. Subsequently, the epitope was more specifically determined using the CLIPS (Chemical Linkage of Peptides onto Scaffolds) technology (Pepscan Presto BV, Lelystad, The Netherlands), in which peptides covering the complete sequence of AMA1 (3D7) were screened. The advantage of using this method in comparison to standard peptide-screening methodology is that the three-dimensional structures of the original protein are simulated by introducing various peptide loop patterns^58^. The results suggest that the antibody specifically binds to the loop of the peptide 194-TLDEMRHFYKDNK-206 within the domain I of AMA1, which is in close proximity to the hydrophobic RON2 binding groove (Fig. 8). The binding site of humAbAMA1 overlaps largely with the epitope of 1F9^56^.

### RON2sp1 and humAbAMA1 compete for binding to AMA1 (3D7)

**Figure 9:**
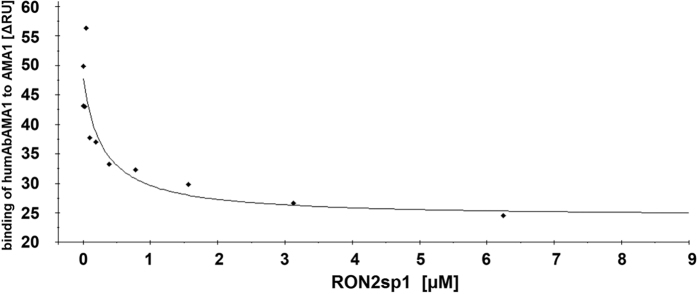
Competition of humAbAMA1 and RON2sp1 peptide. The determination of the epitope region recognized by humAbAMA1 using the peptide array, as well as the binning experiments suggests a possible steric interference between humAbAMA1 and RON2sp1. To investigate the potential competition between RON2sp1 and humAbAMA1 for AMA1-binding AMA1 (3D7) was preincubated with various concentrations of the peptide RON2sp1 for 1 hour. Subsequently, the AMA1 (3D7):RON2sp1 solutions were injected over a surface featuring a defined amount (400 RU) of humAbAMA1 caught on a Protein A-functionalized chip using a BIAcore T200 SPR instrument. For visualization of the results the measured response units were plotted against the concentration of RON2sp1 (μM) used as competitor.

HumAbAMA1 was capable of inhibiting *P. falciparum* 3D7A with an IC_50_ value of 35 μg/mL (Fig. 5) and competed with 1F9 for binding to AMA1 on the same epitope. We were thus interested if humAbAMA1 might as well interfere with the binding of RON2, the natural ligand of AMA1, which had been previously described for both 1F9 and 4G2^42^,^43^,^56^,^59^. AMA1 (3D7) and RON2sp1 peptide, the portion of RON2, which binds to the hydrophobic trough of AMA1^60^, were pre-incubated in different molar ratios. In SPR measurements it was then analyzed to which extent humAbAMA1 could still bind AMA1. As can be seen in Fig. 9 the more RON2sp1 had been binding to AMA1 (3D7), the lower was the proportion of humAbAMA1 recognizing AMA1 (3D7). This indicates that humAbAMA1 binds to an epitope on AMA1 which is at least partially obscured once RON2sp1 has bound to AMA1.

## Discussion

The presented work describes the first human antibody recognizing the malaria vaccine candidate AMA1. HumAbAMA1 reflects a significant degree of affinity maturation *in vivo* (88–96% germ line homology), recognizes a conformational variable epitope lying in very close proximity to the RON2 binding site, and inhibits various *P. falciparum* strains with IC_50_ values ranging from of 35 μg/ml for the strain 3D7A to 195 μg/ml for the strain FCR3.

Most individuals who are premune to malaria have developed antibodies directed at AMA1^10^,^54^,^61^,^62^ and the recognition of conformational determinants of AMA1 is believed to be crucial for a protective AMA1-specific response^63^,^64^,^65^. Rodent antibodies such as 1F9 and 4G2 with inhibitory potential had been reported about fifteen to twenty years ago^66^,^67^. Here, for the first time, a similar human monoclonal antibody is described. The natural affinity maturation of humAbAMA1 resulted in a binding strength of <150 pM, which is close to the range of 10^−11^ M that is considered the lower limit to be achievable *in vivo*^68^,^69^,^70^. Only few human antibodies have been disclosed which possess K_D_ values of as low as ≤10^−10^ M^71^,^72^. Moreover, as compared to 1F9 and 4G2 exhibiting K_D_ values of 1.3 × 10^−9^ M and 3.3 × 10^−9^ M, respectively^73^, the affinity of humAbAMA1 to AMA1 (3D7) is 7- to 18-times stronger.

In analogy to the higher affinity, the inhibitory potential of humAbAMA1 described by its IC_50_ value of 35 μg/ml is well below the IC_50_ values reported for 1F9 (ca. 850 μg/ml)^42^ and AMA1-specific polyclonal human IgG (70–240 μg/ml)^38^,^74^ and to our knowledge, it is the lowest IC_50_-value for a blood-stage antibody described so far. Differences of the values we obtained here and the inhibitory concentrations described in the literature might be based on differences in the assays (parasitemia, hematocrit, readout) as well as the fact that we used recombinant chimeric antibodies, whereas in the publications stated above, the original rodent antibodies have been used.

Very efficient murine mAbs directed at other plasmodial targets such as reticulocyte-binding protein homologue 5 (Rh5) show IC_50_ values of 50–100 μg/ml^44^, and AMA1-specific polyclonal rabbit IgG generated by hyperimmunization protocols could achieve IC_50_ values in a similar range of 34–180 μg/ml^73^,^75^. A direct comparison between polyclonal and monoclonal antibodies is difficult as *in vivo,* an entire spectrum of antibodies contributes to the defense against plasmodial infections and a successful control is likely due to synergistic effects of these antibodies. Therefore, we used humAbAMA1 in combination with humAb10.1, which showed the best IC_50_ value of all MSP10-specific antibodies we described^52^. We found a weak synergistic effect, characterized by a HEWLETT’s S of 1.6. Nonetheless, the effect is inferior to the synergistic effect that has been described for other polyclonal antibody combinations, *e.g.* murine Rh4- and Rh5-specific mAbs^76^, or combinations of anti-plasmodial chemotherapeutics^77^,^78^.

Although humAbAMA1 inhibits the parasite strains HB3 and FCR3 with IC_50_- values of 126 and 195 μg/ml, which is 3.6 to 5.6-fold higher than the respective IC_50_-value measured in the strain 3D7A, it is still superior to the inhibitory capacity of the well-described antibodies 4G2 and 1F9. It is a very interesting observation that the antibody showed a direct relation between the affinity to the antigen variant and the inhibitory capacity to the respective parasite strain. This needs to be reconfirmed with an increased number of parasite strains and antigen variants.

AMA1-specific antibodies may inhibit plasmodia by different means, *i.e.* (1) the blocking of epitopes that are important for the interaction with other proteins such as RON2, (2) the prevention of structural rearrangements within the AMA1 molecule required for efficient RON2-binding, (3) the impairment of the proteolytic processing of AMA1, and (4) the interference of the redistribution of the cleavage products^79^. Moreover, a fourth mode of action could be the premature induction of the phosphorylation of serine 610 in the cytoplasmic tail of AMA1 and the untimely initiation of downstream signalling events, *e.g.* by imitating the binding of its natural ligand. The phosphorylation of S610 was shown to be critical for invasion^37^,^80^,^81^. Based on the current data at hand we cannot conclusively say how humAbAMA1 exerts its inhibition of *P. falciparum* 3D7A. However, there are several clues. The first evidence is that 1F9 and humAbAMA1 competed for binding to AMA1 (3D7), whereas 4G2 and humAbAMA1 does not (Fig. 7). 4G2 binds to a region of domain II which is distant from the hydrophobic trough of AMA1^34^,^43^, but until now, the crystal structure of 4G2 binding to AMA1 could not be resolved. 1F9 on the other hand binds close to the hydrophobic cleft of AMA1 and to adjacent regions in domain I, including residues P188, M190, P192, E197, H200, F201, Y202, D204, M224, I225, N228 in AMA1 (3D7)^56^. This region shares a significant overlap with the epitope region of humAbAMA1 identified by peptide array analysis (AA 194–206). Additionally, we showed either a clear intereference with the binding site of RON2, since humAbAMA1 could not bind efficiently to AMA1:RON2sp1 complexes in an SPR setup (Fig. 9). This implies that by the binding of RON2sp1 the epitope of humAbAMA1 was either masked or structurally rearranged by RON2sp1. The evidence given strongly argues for an interference of humAbAMA1 with the binding of RON2 to AMA1 on the parasite, thus preventing the formation of the AMA1:RON2 complex, leading to the inhibition we observed in GIAs. However, this mode of inhibition remains to be proven.

In this study we took a closer look at *P. falciparum* strains 3D7A, HB3 and FCR3 as well as their respective AMA1 alleles. It is well known that AMA1 is subject to diversifying and balancing selection in order to evade the host’s immune system as well as to maintain its structure and function^82^,^83^,^84^. Therefore, future experiments will have to address if humAbAMA1 is capable of inhibiting even more *P. falciparum* strains than the presented ones. Nevertheless, the data presented here already shows that the antibody has some cross-strain inhibitory activity, even though it binds to a variable region of the parasite. The determination of the structures of a Fab fragment of humAbAMA1 and of the Fab:AMA1 complex will likely help to shed more light on the nature of the epitope and the mode of action of the new antibody we presented.

Since transient expression in *N. benthamiana* is a well-established antibody^52^ and vaccine candidate^85^ expression platform in our lab, we used it for the production of humAbAMA1 and compared expression levels and antigen-binding properties with humAbAMA1 produced by transient transfection of HEK293-6E cells. As shown by SPR-analysis, the plant and HEK produced molecules show no significant differences in AMA1 binding.

Besides elucidating the epitope targets and parasite growth inhibitory mechanisms of naturally occurring human *Pf*-specific mAbs, such molecules are also of interest for therapeutic use. Of crucial importance for a therapeutic antibody is its ability to inhibit a brought set of parasites occurring naturally. The set of parasites chosen here represent a number of variants of the estimated epitope. All strains could be inhibited by humAbAMA1, albeit at varying concentrations. Nevertheless, further investigations are necessary in order to estimate the full therapeutic potential of humAbAMA1. Even though the majority of monoclonal antibodies used in therapy is targeting tumors or chronic inflammatory diseases, there are certain applications for antibodies and antibody cocktails also in other fields like the treatment of toxin mediated^86^ and infectious diseases. Post-exposure treatment of rabies infections includes the injection of rabies virus specific antibodies isolated from horse serum^87^, but various groups are working on the development of recombinantly produced humanized or human monoclonal antibodies to replace such sera. Humanized monoclonal antibodies have been proposed as therapeutics for West Nile Virus infections^88^ and a cocktail of three humanized antibodies^89^ targeting the Ebola virus EBOV glycoprotein has received some attention in the context of the latest outbreak of EBOV in several West African countries. The antibody-based Ebola-emergency treatment ZMapp used in a small number of human patients has been produced in *N. benthamiana* plants by transient expression^90^. In the case of malaria antibodies could be used for short-term prevention and/or therapy in clinical cases with drug resistant strains^91^. Based on antibodies such as the one presented here in combination with affordable expression systems, the cure of malaria infections or the generation of passive vaccination strategies becomes feasible.

## Materials and Methods

### Ethics statement and blood donors

This study was conducted according to the principles expressed in the Declaration of Helsinki. Ethical approval was given by the Committee on Human Research Publication and Ethics (CHRPE) of the Kwame Nkrumah University of Science and Technology in Ghana. After the goals of the study had been carefully explained, written informed consent was obtained from all participants. By the time of blood donation, the 31 volunteers were between 20 and 45 years old and had not undergone any clinical phase of malaria for at least two years. The Ghanaian study, as well as the inclusion and exclusion criteria had been described previously^92^. Each volunteer donated 50 ml of heparinized blood which was then used for the preparation of plasma and PBMCs as described below.

### Preparation of peripheral blood mononuclear cells (PBMCs) from Ghanaian donors

PBMCs were prepared from 50 ml of heparinized peripheral blood from each donor. The blood was diluted in one volume of PBS and was then layered onto Ficoll (GE Healthcare, Uppsala, Sweden). After centrifugation (800× *g*, 20 min, 25 °C, without break) the PBMCs in the interphase were drawn off, washed once in PBS containing 1% (v/v) FCS (400× *g*, 10 min, 25 °C), and once in PBS containing 10% (v/v) FCS (300× *g*, 10 min, 25 °C). They were then taken up in freezing medium (10% (v/v) DMSO in FCS) at cellular concentrations ranging from 3–6 × 10^6^ cells/ml and were cryopreserved. The temperature was gradually decreased to −80 °C (1 K/min) using isopropyl-containing freezing containers.

### Fluorescent labeling of recombinant AMA1 (DiCo 1-3)

AMA1 (DiCo 1-3) was kindly provided by Jürgen Drossard (Fraunhofer IME, Aachen, Germany) with the kind permission of Ed Remarque (BPRC Rijswijk, The Netherlands). The proteins were chemically labeled with “AlexaFluor488-TFP” (Life Technologies, Darmstadt, Germany) as recommended by the manual’s instructions. On average, the proteins were found to be labeled with 5.9 AlexaFluor 488 moieties (mol/mol) as determined by a spectrophotometric measurement.

### Reduction and alkylation of AMA1 (3D7)

For testing of conformational dependency of epitope recognition by polyclonal AMA1-specific antibodies and by humAbAMA1, AMA1 (3D7) was irreversibly reduced. To this end, it was treated with 5 mM DTT at 56 °C for 45 min while shaking. The reaction was stopped by adding 15 mM iodoacetamide. After incubation for 30 min in the dark 5 mM DTT were added to quench excessive iodoacetamide.

### Flow cytometric cell sorting

PBMCs were thawed gently (37 °C) and were washed once with RPMI 1640 medium (10% (v/v) FCS). The staining with human IgG- and human CD22-specific antibodies (anti-IgG-PE and anti-CD22-APC, BD Biosciences, Heidelberg, Germany) and fluorescent AMA1 (DiCo 1-3) in labeling buffer (2% (v/v) FCS, 1 mM EDTA in PBS (137 mM NaCl, 2.7 mM KCl, 8.1 mM Na_2_HPO_4_, 1.5 mM KH_2_PO_4_)) was carried out on ice for 30 min. Cells were then washed three times with cold labeling buffer and were individualized with a 30 μm-mesh before resuspension in particle-free RPMI 1640 (20% (v/v) FCS). Antigen-specific IgG^+^/CD22^+^ B cells were sorted with an FACS Influx cytometer (BD Biosciences, Heidelberg, Germany) into RPMI 1640 (20% (v/v) FCS).

### Epstein-Barr virus transformation

After a viability staining with Trypan blue specific IgG^+^/CD22^+^ B lymphocytes were infected with Epstein-Barr virus (B95-8) and subjected to virocrine transformation as described^52^,^93^.

### Enzyme-linked immunosorbent assays

ELISA procedures for the various parts of the study were carried out as described before to: (A) identify promising B cell cultures^52^, (B) assess the specificity of recombinant human antibodies^52^, and (C) screen the Ghanaian plasma samples for reactivity against AMA1 (3D7)^54^.

### Rescue and cloning of immunoglobulin variable regions

cDNA of promising cultures was generated with the “SuperScript III CellsDirect” kit (Life Technologies, Darmstadt, Germany) according to the manufacturer’s instructions using oligonucleotides for the rescue and cloning of human immunoglobulin variable regions (Vh and Vl) as previously described^94^. Primers for the cloning into pTRAkt vectors were designed to correct any mutations introduced by the degenerate primer set used for nested PCRs – thereby reconstituting the authentic sequences of 1E4 Vh and 1E4 Vl. Vh and Vl were cloned individually into the respective pTRAkt vectors for apoplastic expression of human full-size IgG1:κ antibodies in plants^95^.

### Expression of full-size humAbAMA1 IgG in *Nicotiana benthamiana*

Expression and purification of full-size IgG by *Agrobacterium*-mediated transient transfection of *N. benthamiana* plants was performed using agrobacteria transformed with the vectors pTRAkt_IgG1_H_1E4 (humAbAMA1 IgG heavy chain) and pTRAkt_Igκ_1E4 (humAbAMA1 Igκ light chain), respectively, as described elsewhere^95^.

### Expression of full-size humAbAMA1 IgG in HEK293-6E

Expression of humAbAMA1 in HEK293-6E cells was performed with the help of vector pTT5, into which the expression cassette used in pTRAkt vectors described above was inserted. Transfection, cultivation of the cells as well as ProteinA-based purification was performed as described before^96^.

### *Plasmodium falciparum* culture and growth inhibition assays

*P. falciparum* strains 3D7A, HB3 and FCR3 were cultured as described^97^. Parasites were maintained at a hematocrit of 5% in RPMI 1640, 25 mM HEPES, 2 mM L-glutamine, 50 μg/ml gentamycin, 10% Albumax II.

*In vitro* growth inhibition of the various antibodies was assessed with *P. falciparum* 3D7A, HB3 and FCR3 as described before^53^. HumAbAMA1 was applied in concentrations ranging from 4.35 mg/ml to 4.25 μg/ml. Polyclonal rabbit-anti-AMA1 IgG (BG98) in a concentration of 6 mg/ml was taken as a positive control^98^. Serum IgG of a naïve rabbit was taken as a corresponding negative control (6 mg/ml). HumAb 2G12, an HIV-1-specific IgG, was taken as a plant-expressed non-specific control. 2G12 was kindly provided by Jürgen Drossard. Prior to the setup of the assay *P. falciparum* strains had been synchronized three times by sorbitol treatments^99^. Parasites were seeded at schizont stage at a parasitemia of 0.4% in 2% hematocrit. After an incubation period of 40–46 h, the GIA was harvested and frozen at −80 °C for at least 16 h. Parasitaemia was read out by assessing the pLDH activity^100^. Relative growth inhibition was calculated as follows: % inhibition = 100 − 100 × [A_655nm_ (sample) − A_655nm_ (erythrocyte control)]/[A_655nm_ (schizont control) − A_655nm_ (erythrocyte control)]. In order to test for a synergy of invasion inhibition both humAbAMA1 and a MSP10-specific antibody, humAb10.1 (described in ref. 52), were employed. To this end, the antibodies were used singly and in ratios proportional to their respective IC_50_ values. The ratios were 1:1, 1.5:1, 4:1, 1:2, and 1:4. Data were analyzed by calculating HEWLETT’s S and the sum of FIC, a quantitative index of the extent of synergy^76^.

### Immunofluorescence assay

For immunofluorescence assays *P. falciparum* 3D7A was synchronized by sorbitol treatments. Parasitized erythrocytes were washed once with RPMI 1640 before being taken up in FCS (low bovine IgG) and smeared onto object slides. Parasites were air-dried and then fixed in methanol at −20 °C for 10 min. Slides were incubated with 5 μg/ml humAbAMA1 and 20 μg/ml mouse-anti-MSP4(EGF) (mAb 2.44, kindly provided by Alexander Boes, Fraunhofer IME, Germany)^95^ in 1% FCS (low bovine IgG) in PBS in a moist box at 25 °C for 1 h. After five washing steps in PBS, the slides were treated with 1:100 goat-anti-mouse IgG(H + L)-AlexaFluor488 (Life Technologies, Darmstadt, Germany), 1:1000 goat-anti-human IgG(H + L)-Cy3 (Jackson ImmunoResearch/Dianova, Hamburg, Germany), and 10 μg/ml Hoechst 33342 in a moist box at 25 °C for 1 h. Five washing steps were carried out. Mounting medium (“ProLong Gold Antifade Reagent”, Life Technologies, Darmstadt, Germany) was used to conserve the staining. Images were taken with a Leica TCS SP8 microscope at a 630-fold magnification.

### Surface plasmon resonance spectroscopy

In our study SPR measurements fulfilled three purposes. (A) Determination of the affinity of humAbAMA1 to AMA1 (3D7), (B) pairwise epitope mappings with monoclonal rodent antibodies 1F9 and 4G2, and (C) RON2-competition measurements. All measurements were performed by using a Biacore T200 instrument (Biacore, GE Healthcare) and CM5-S-Series sensor chips at 25 °C with HBS-EP (10 mM 4-(2-hydroxyethyl)-1piperazineethanesulfonic acid, 150 mM NaCl, 3 mM EDTA, 0.005% (w/v) polysorbat-20) as running buffer. (A) The kinetic analysis of the humAbAMA1 interaction was performed as previously described^73^. (B) To roughly map the epitope of humAbAMA1 in relation to the binding sites of previously described AMA1 specific monoclonal antibodies 1D7, 1F9 and 4G2, AMA1 (3D7) was captured (1600 RU) using a CM5 surface functionalized by EDC-NHS chemistry with 7000 RU of the rat monoclonal 1D7. Mab1D7 is specific for the ectodomain III of AMA1, recognizes an epitope, which is distant from the hydrophobic cleft, and thus does not interfere with 1F9 or 4G2 binding. The antibodies 1F9, 4G2 and humAbAMA1 (at concentrations of 1 μg/ml and/or 10 μg/ml) were separately and consecutively injected for 180 s and binding levels were compared. (C) In order to rule out if humAbAMA1 and RON2 compete for binding to AMA1, AMA1 (3D7) and RON2sp1 were mixed in different molar ratios and incubated for 1 h at 25 °C. (RON2sp1 corresponds to the portion of RON2 which binds to the hydrophobic through of AMA1^102^). The extent to which humAbAMA1 could bind to AMA1:RON2sp1 complexes was then determined. To this end, first, humAbAMA1 was caught on a Protein A-functionalized chip and then the different pre-incubated AMA1:RON2sp1 solutions were injected.

### Epitope mapping using Pepscan screening

The epitope mapping of humAbAMA1 was carried out by Pepscan Presto BV (Lelystad, The Netherlands) as described before^58^. For the screening, four sets of peptides were used, all based on the AMA1 sequence of the parasite strain 3D7. Set 1 consisted of linear peptides of the length of fifteen amino acids. Set 2 consisted of constrained peptides of the length of 17 amino acids, with cysteine residues inserted at positions 1 and 17, and amino acids 2–16 representing AMA1 sequences. Cysteine residues on positions 1 and 17 were linked using the CLIPS technology as described^58^. Set 3 was designed to mimic α-helices of AMA1. These peptides consisted of 16 amino acids with cysteine residues inserted at positions 1 and 5. Set 4 constituted peptides with lengths of 22 amino acids and with cysteine placed on position 1 and 22. In this peptide set 4, amino acids on positions 11 and 12 had been replaced by proline and glycine, respectively. In all non-linear peptide sets described, cysteine residues occurring naturally the AMA1 sequence were chemically protected by acetamidomethyl in order to prevent unintentional reaction.

## Additional Information

**How to cite this article:** Maskus, D. J. *et al*. Characterization of a novel inhibitory human monoclonal antibody directed against *Plasmodium falciparum* Apical Membrane Antigen 1. *Sci. Rep.* **6**, 39462; doi: 10.1038/srep39462 (2016).

**Publisher's note:** Springer Nature remains neutral with regard to jurisdictional claims in published maps and institutional affiliations.

## Figures and Tables

**Table 1:** Surface plasmon resonance measurement results for antibody affinity against AMA1 variants of *P. falciparum* strains 3D7, HB3 and FCR3.

Antigen AMA1 strain variant	Antibody	K_a_ Association rate (±SE)	K_d_ Dissociation rate (±SE)	K_D_ Affinity constant (±SE)
3D7	humAbAMA1 (HEK293-6E)	3.05 × 10^5^ (±2.8 × 10^2^) M^−1^ sec^−1^	3.23 × 10^−5^ (±1.6 × 10^−6^) sec^−1^	106 (±5) pM
	humAbAMA1 (*N. benthamiana*)	3.28 × 10^5^ (±1.4 × 10^2^) M^−1^ sec^−1^	4.44 × 10^−5^ (±2.6 × 10^−6^) sec^−1^	135 (±8) pM
HB3	humAbAMA1 (*N. benthamiana*)	5.46 × 10^5^ (±1.3 × 10^3^) M^−1^ sec^−1^	3.64 × 10^−4^ (±3.9 × 10^−6^) sec^−1^	667 (±7) pM
FCR3	humAbAMA1 (*N. benthamiana*)	1.17 × 10^5^ (±2.3 × 10^2^) M^−1^ sec^−1^	8.52 × 10^−3^ (±8.9 × 10^−6^) sec^−1^	73 (±0.2) μM

**Table 2:** *In vitro* growth inhibitory activity (expressed as IC_50_) of the human and chimeric AMA1-specific antibodies against *P. falciparum* strains 3D7A, HB3 and FCR3.

Parasite strain	3D7A	HB3	FCR3
Antibody			
	**IC_50_ (95% Confidence Interval)**		
humAbAMA1 (*N.b.*)	35 (33–37) μg/ml	126 (112–141) μg/ml	196 (159–239) μg/ml
humAbAMA1 (HEK293-6E)	35 (34–37) μg/ml	ND	ND
4G2 (*N.b.*)	105 (99–111) μg/ml	396 (362–433) μg/ml	817 (674–990) μg/ml
1F9 (*N.b.*)	292 (250–342) μg/ml	>1750 μg/ml	1438 (1233–1676) μg/ml

ND: Not determined; N.b.: *Nicotiana benthamiana*.
